# A Tagless Indoor Localization System Based on Capacitive Sensing Technology

**DOI:** 10.3390/s16091448

**Published:** 2016-09-07

**Authors:** Alireza Ramezani Akhmareh, Mihai Teodor Lazarescu, Osama Bin Tariq, Luciano Lavagno

**Affiliations:** Politecnico di Torino, Corso Duca degli Abruzzi, Torino 24-10129, Italy; mihai.lazarescu@polito.it (M.T.L.); bintariq.osama@ismb.it (O.B.T.); luciano.lavagno@polito.it (L.L.)

**Keywords:** capacitive sensing, indoor localization, tagless localization

## Abstract

Accurate indoor person localization is essential for several services, such as assisted living. We introduce a tagless indoor person localization system based on capacitive sensing and localization algorithms that can determine the location with less than 0.2 m average error in a 3 m × 3 m room and has recall and precision better than 70%. We also discuss the effects of various noise types on the measurements and ways to reduce them using filters suitable for on-sensor implementation to lower communication energy consumption. We also compare the performance of several standard localization algorithms in terms of localization error, recall, precision, and accuracy of detection of the movement trajectory.

## 1. Introduction

Reliable indoor person location is essential for several location-based services that improve life quality and safety, such as automatic control of lighting, heating or home electronic devices based on person presence and physical location [1]. Indoor localization also provides an effective means to track the position, motions and reactions of a patient, an elderly person or any person with special needs for medical observation or accident monitoring for assistive health care [2]. Daily routine tracking can effectively detect abnormal behaviors, such as waking up too late or staying in the same position longer than expected, which may be due to deteriorating health conditions.

For wide adoption, the sensing technique should be tagless, passive, privacy-aware and unobtrusive. Tag-less is important since people, especially elderly, often forget or are reluctant to wear a tag or device, or to accept intrusive monitoring of their daily routine.

Unlike active systems, passive systems do not require the person to explicitly interact with the system in order to be localized. For instance Lively [3], an active system, requires the person to move objects instrumented with sensors (e.g., a pill box or a fridge door), otherwise they lose track of location and activities. This may limit the system reliability and acceptance.

Image-based systems [4] raise privacy concerns. Most persons do not wish to be monitored by a camera, even when they are assured that the system will store just blurred shapes of the person in its database [5].

Lastly, the system should not interfere with the daily activities of the person, for example, the movements. A hidden, non-intrusive system is highly desirable, even more so for security and surveillance applications, where apparent sensors are easier to disable.

Several systems have been proposed for human location sensing [1]. However, each has specific limitations in the scenarios described above. For instance, pressure sensors have about 5 cm accuracy [1] and can be concealed under the floor surface, but the deployment cost can be very high for a large area, mostly because of the required modifications of the floor surface.

Infrared sensors can also be used. The Canary system [6] and eLea [7] use pyroelectric infrared sensors (PIR) sensors, which are inexpensive and easy to install. In [8], the authors use the analogue output of a set of PIRs to localize and identify human subjects in a designated area. However, PIR-based sensors detect only movements and are ineffective for monitoring still activities like sleeping, reading, or watching TV. Moreover, they operate only withing unobstructed line of sight and they are sensitive also to non-human heat sources, such as sun and stoves, which increase the chance of false detection.

Several sensing solutions use data fusion from a variety of sensor types to increase the accuracy and resolution in assisted living application scenarios [9].

Capacitive sensing is a technology that allows the detection and tracking of conductive and non-conductive objects [10]. It has complementary characteristics to the techniques described above, as well as several unique advantages. Hence, it can be used for indoor location and movement detection both on its own and in combination with, e.g., the largely complementary PIR sensors.

Capacitive sensors use capacitive transducers that can operate in three different modes (or configurations). Zimmerman et al. [11] introduced in 1995 the transmit and shunt operation modes. For example, the authors of [12,13] use these two operation modes for indoor human body detection. However, since transducers operating in these two modes require at least two galvanically coupled plates, their use would significantly increase the complexity and the cost of the installation for our application domains.

**Figure 1:**
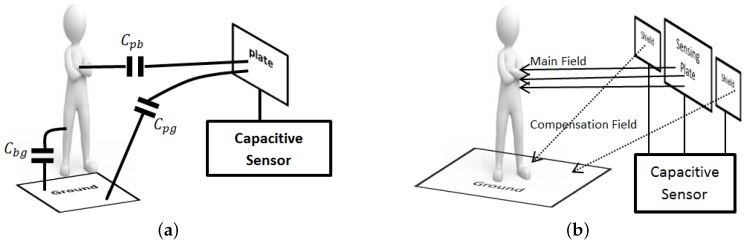
(**a**) Main sensor capacitances in load mode: plate-body ($C_{\mathrm{pb}}$), plate-ground ($C_{\mathrm{pg}}$), and body-ground ($C_{\mathrm{bg}}$); (**b**) Use of compensation fields for short-range load-mode capacitive sensors to reduce $C_{\mathrm{pg}}$.

In 1998, Smith et al. [14] introduced the load operation mode for capacitive transducers. This mode uses the human body as a constant-potential plate (Figure 1a) and requires just one-plate transducers, which are much more suitable for deployment in our application domains. However, the sensing range of the existing load mode capacitive sensors is comparable to their plate dimensions, which is generally too short for our applications of interest.

In this article we propose a method that significantly extends the sensing range of load-mode capacitive sensors in order to allow them to sense a person at distances suitable for localization within the area of interest (e.g., one or more rooms).

We report how we designed and characterized an indoor localization system for persons using capacitive sensing techniques. We also demonstrate, using different figures of merit such as precision and recall, how promising our method is in a realistic indoor localization scenario.

The rest of the paper is organized as follows. Related work on capacitive sensing for indoor localization is discussed in Section 2. In Section 3 we discuss our basic strategy for capacitive sensing. In Section 4 we illustrate the building blocks of our system, along with a detailed explanation for each constituting unit. In Section 5 we discuss the filtering schemes that we have used and the localization algorithms. The experimental results are discussed in Section 6. Section 7 concludes the paper and presents future research directions.

## 2. Related Work

In [13,15,16,17], the authors introduce a position and fall detection system based on capacitive sensing. They integrate the sensing electrodes into the floor surface (in [17] they add an extra plate to the ceiling). In order to be localized, the person needs to step on the different floor areas equipped with sensing electrodes. The main drawback of these systems is that they are hard to install and maintain. The cost to cover large areas can rise quickly.

Togura et al. [18] designed a capacitive sensing prototype based on a delta-capacitance-voltage converter, to detect the presence of the driver on the seat and control some actuators inside the cabin. The authors claim that they can detect a human hand up to 30 cm at best. However, the range of their solution is too short to be useful for indoor localization purposes, which require sensing ranges beyond 1 m.

Electric and electromagnetic noise and nearby conductive objects can also interfere with the operation of the capacitive sensors and limit their accuracy and/or sensing range. Wimmer et al. [12] designed Thracker, a capacitive sensor working in load mode for human gesture recognition. It senses the human presence in front of a display and allows the person to interact with it by using as sensing electrodes four plates, each 4 cm wide, on the four corners of the display. They use a capacitance-frequency converter based on a resonant circuit including a 555-based timer multi-vibrator, whose output frequency varies with the distance between the sensor electrodes and the hand. However, the accuracy of the sensing degrades quickly when the distance between the sensor plates and the human body increases. Also, metallic nearby objects can easily dampen their sensed signal and reduce the effective sensing range down to about 20 cm. This is a general problem for all solutions based on capacitive sensing, including ours, thus we discuss at length the resulting signal processing requirements.

In [10,19,20], the authors propose a capacitance-frequency transducer for human body detection in which the sensor plate is actively guarded by an auxiliary field that reduces the unwanted coupling effects between the nearby objects and the sensor plate (Figure 1b). The authors claim that the usable sensor range is about two or three times the diameter of the electrode. They also mention some criteria to find the maximum achievable range, such as the acquisition time and properties of the environment. They claim to be able to detect a human body up to 100 cm using a 10 cm × 10 cm sensor plate at best. However, the method to shield high sensitivity load-mode capacitive sensors from the presence of nearby conductive bodies cannot be extended to longer ranges, because the integrity of the auxiliary field degrades rapidly with the distance. The range decreases even further for smaller sensor plates.

MacLachlan [21] demonstrates the prototype of a capacitive proximity sensor for human presence based on a spread spectrum technique with a range of about 1 m. However, the author does not mention any realistic localization results using this system. Moreover, the system cannot detect the absolute location of a person.

### Our Main Contributions

To improve the long-range accuracy of capacitive sensors for our target application domains, we propose complementary techniques that are effective over longer sensing ranges than those discussed above. The capacitive sensor that we propose for tagless long-range sensing of the human body is made of a capacitive transducer operating in load mode, with a novel combination of several data processing techniques that are aimed to significantly increase the sensing range. The ranges of these new capacitive sensors are thus able to satisfy the needs of low-cost applications that require tagless low-power indoor human presence monitoring, for which the existing load-mode or infra-red sensors cannot be used.

Briefly, the operation of the system consists of:

1. indirect measurement of the capacitance of the transducer by measuring the frequency of a relaxation oscillator whose electrical and timing characteristics depend on transducer capacitance;
2. use of baseband digital filters to attenuate the noise captured by the sensor;
3. use of localization algorithms to infer the position of the person using the data from several load-mode capacitive sensors within one or several rooms.

In this article, we will assume that a single person is moving inside the area of interest (e.g., a room or an apartment). We leave to future work how to locate and, if possible, discriminate between different persons. For our main area of application, which is the assisted living of the elderly people, the case of a single person is by far the most interesting one.

## 3. Capacitive Sensor

Electrical capacitance is defined as the electrical charge stored on a conductive object divided by the resulting change of its potential. Thus, the capacitance of an object depends primarily on its size, the distance to other objects, and the dielectric properties of the objects and of the dielectric between them (e.g., air) [19]. The capacitance of a parallel-plate capacitor with plate areas significantly larger than plate distance is given by Equation (1)

(1) $$C=\frac{\varepsilon_{0}kA}{d}$$

where *C* is the total capacitance formed between the two plates (in Farad), *k* is the relative dielectric permittivity of the material between plates (k=1 in case of free space), $\varepsilon_{0}$ is the absolute dielectric permittivity of free space ($8.854\times 10^{-12}$ F/m), and *d* is the distance between the capacitor plates (in meters). We discuss later that similar laws govern also the capacitance of non-planar distant objects.

Person localization based on capacitive sensing measures indirectly the variations of *d*. With several sensors (at least three in a typical scenario) it can determine the person position. The sensitivity (to both signal and environmental noise) depends especially on *A*, the effective area of capacitor plates.

Our capacitive sensor uses a transducer operating in load mode whose capacitance varies based on the distance to a nearby human body and other objects (as shown in Figure 1a). We reduced the influence of the latter via several signal processing techniques and we determined experimentally that the sensitivity of the capacitance to distance decreases steeply at longer distances.

### Measurement Results

**Figure 2:**
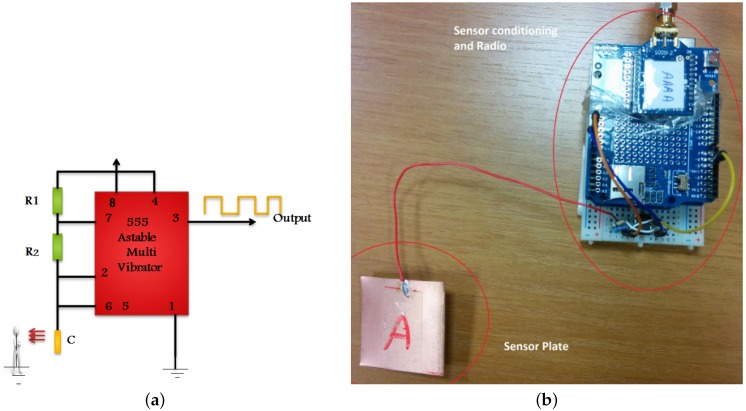
(**a**) The 555-based capacitance-frequency converter; (**b**) A sensor prototype that uses a copper-clad PCB as capacitive transducer.

To measure the capacitance change with the distance in a real environment, we used a relaxation oscillator based on a 555 timer integrated circuit (IC) configured in astable mode (it oscillates indefinitely), as shown in Figure 2a.

The output frequency of the oscillator is determined by Equation (2)

(2) $$\mathrm{Frequency}=\frac{1}{0.7(R_{1}+2R_{2})C}$$

With $R_{1}=200\;k\Omega$ and $R_{2}=560\;k\Omega$, the oscillation frequency was around 70 kHz for a 4 cm × 4 cm plate.

For the experiment, we attached first a 4 cm × 4 cm plate to the oscillator circuit (shown in Figure 2b) and a person stood at a distance of 2 m in front of it, facing the plate. Every 20 s the person moved closer to the sensor plate in 20 cm steps up to a distance of 40 cm, and in 10 cm steps afterwards.

At each distance, we gathered 20 frequency samples, one per second. The frequency was measured by counting the full periods of the oscillator in an interval of one second, using an Arduino board. The raw samples were wirelessly sent to a computer (base station) using a Zigbee module, for postprocessing.

**Figure 3:**
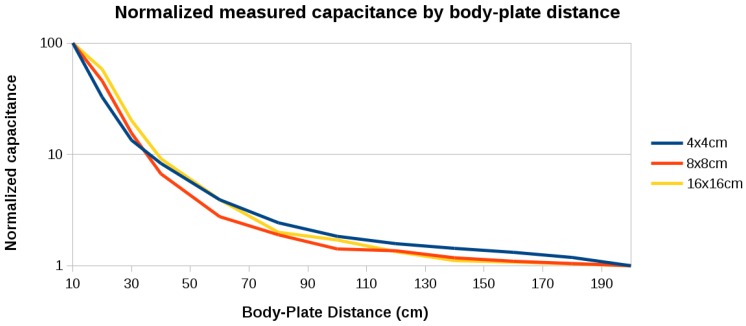
Normalized measured capacitance by body-plate distance using a 4 cm × 4 cm, 8 cm × 8 cm, and a 16 cm × 16 cm plate.

**Figure 4:**
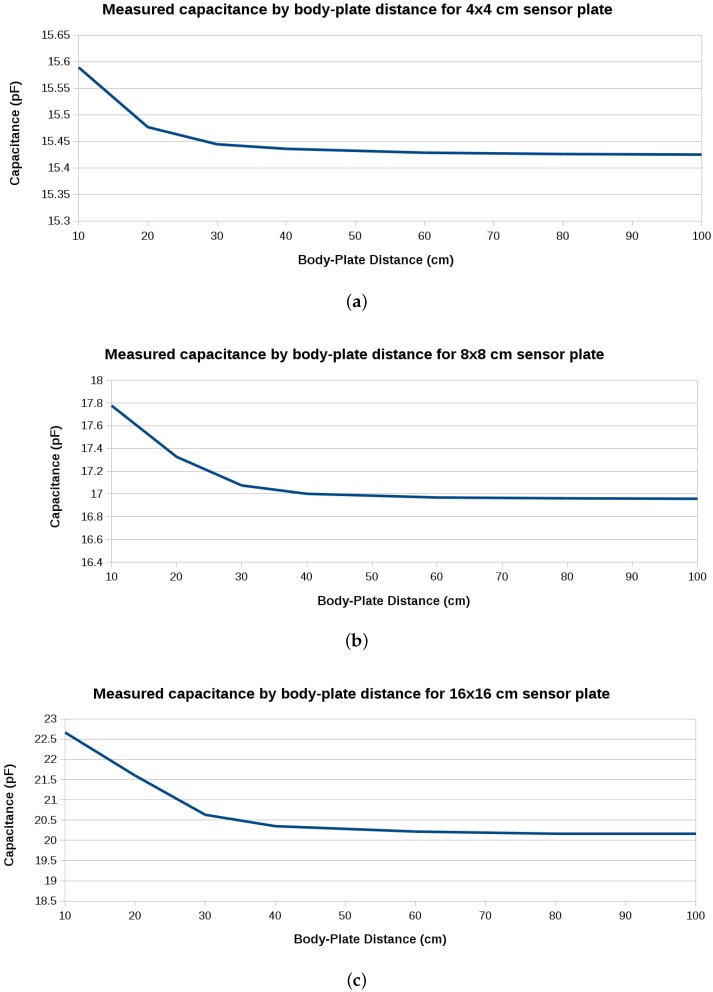
Measured capacitance versus body-plate distance using (**a**) 4 cm × 4 cm; (**b**) 8 cm × 8 cm; and (**c**) 16 cm × 16 cm plates.

The 20 frequency samples per position were averaged and the total capacitance (plate-body capacitance plus plate-ground capacitance) was calculated using Equation (2). Figure 3 shows the results normalized with respect to the capacitance of each sensor when the person is at 2 m, then at 10 cm distance from it. Figure 4 shows the raw capacitance values, which reflect the variation of the sensitivity with plate size. As expected, the 16 cm × 16 cm plate has the best sensitivity.

The measurement of the capacitance is affected by various types of environmental noise, such as noise generated by nearby appliances, static charges, switching supplies, radio waves, and temperature and humidity variations. Hence, we need to improve the signal to noise ratio of the sensor using some filtering techniques, which we discuss in Section 5.

## 4. Sensing System Architecture

In order to detect and localize a single person indoors, we use several statically mounted sensors in the environment, e.g., on walls or near locations of interest (armchair, sofa, bed, bathroom or kitchen sink). The multi-dimensional data collected by them can be used to estimate the position of the person using a variety of methods, as discussed in Section 5.2.

**Figure 5:**
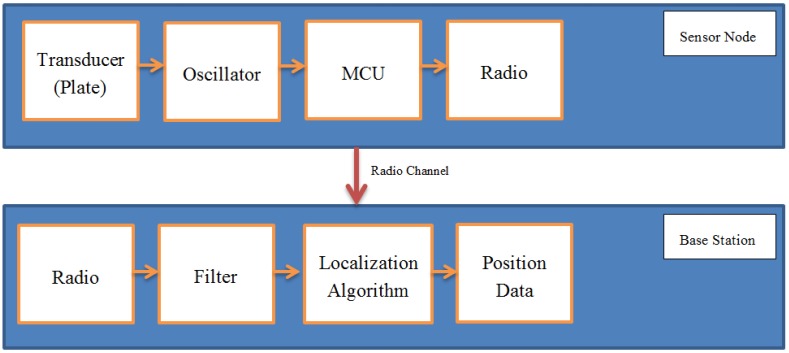
Main building blocks of the detection system, at sensor and base station level.

Figure 5 shows the architecture of the system. As the person moves, the capacitance of sensor transducers changes with their distance to the body. Capacitance variations are converted on the sensor to frequency variations using a capacitance-controlled oscillator (Figure 2a). As the person comes closer to a sensor plate, the mutual capacitance ($C_{\mathrm{pb}}$ in Figure 1a) increases and the oscillator frequency decreases according to Equation (2). The micro-controller (MCU) of the sensor measures the oscillator frequency with a rate that will be discussed below and sends it to the base station using a Zigbee radio module. Note that the sensor is battery-powered and has no galvanic connection to the ground. This is a realistic setting whose effect on the measurements will be discussed next.

Through its Zigbee radio receiver, the base station receives a continuous stream of raw frequency measurements from each installed sensor. The received streams are forwarded by the base station MCU to a USB-connected personal computer for further processing.

### 4.1. Experimental Setup

The data from the sensors, as will be shown next, can be affected to two main types of noise:

- high-frequency noise (i.e., close to our sampling frequency of 1 Hz), from appliances and light switches. We reduce these using low-pass filters;
- a low-frequency drift, where the DC component varies very slowly by a significant amount (larger than the useful signal component), due to slow leak of static charges, temperature and humidity changes, and so on. We reduce these using high-pass filters.

#### 4.1.1. Long-Term Tracking Experiment

**Figure 6:**
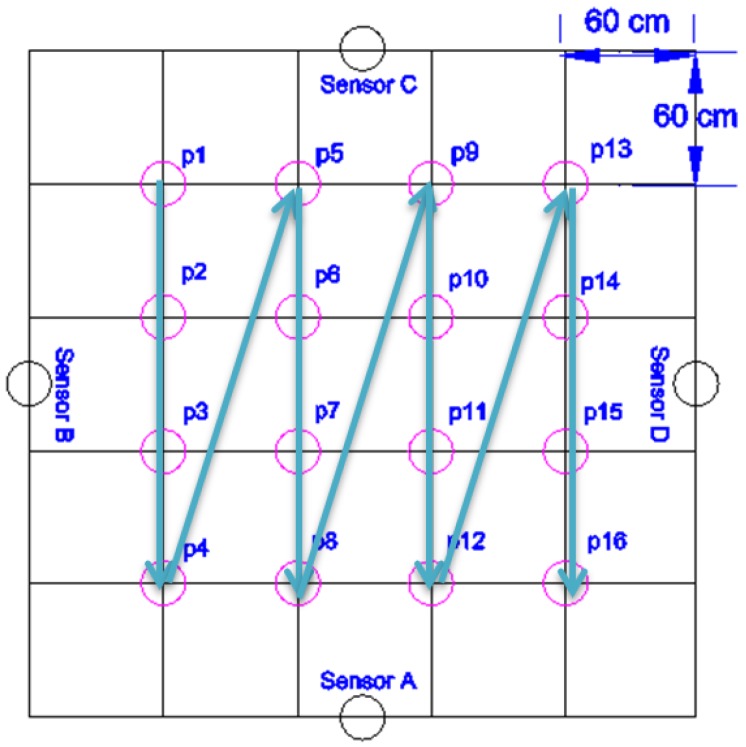
Room layout, sensor placement, room positions and the walked path for localization algorithm training (thick arrows).

We performed all our experiments in a 3 m × 3 m laboratory area that we designated as “the room”, shown as the outer perimeter in Figure 6.

This is the size of a typical bedroom or of a small living room in many European and Asian countries. Larger rooms can have sensors attached also to tables, sofas, and so on.

**Figure 7:**
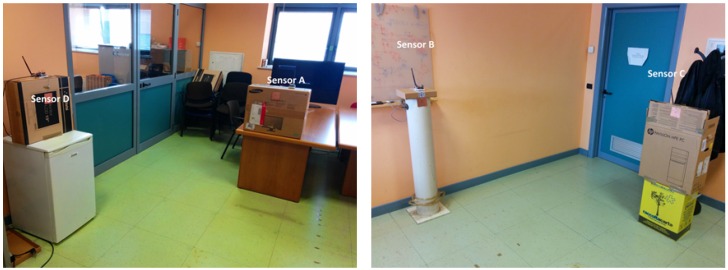
Sensor A, B, C and D placement in the experiment room.

In this room we placed a capacitive sensor in the middle of each wall (shown as sensor A, sensor B, sensor C and sensor D in Figure 6). On the floor of the room we defined a grid with four rows and four columns with a 60 cm step, which gives the 16 positions shown in Figure 6. Considering that our room is not surrounded by real walls, we used cardboard boxes to support the sensors as shown in Figure 7.

The first experiment, referred to as “long-term experiment”, consisted in monitoring the location of the person in the room over a period of about 20 min. The person stood in each one of the 16 room position for 80 s. This provided 1280 samples in total, plus 80 samples while the person was outside of the room. In each position, the person rotated counterclockwise every 20 s (thus 4 times), each time by 45 degrees.

**Figure 8:**
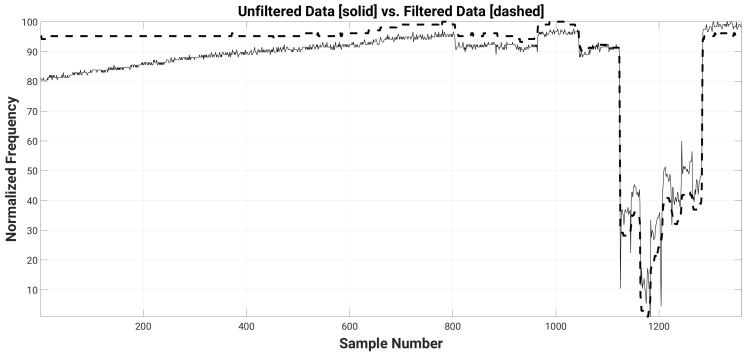
Long-term experiment raw data (solid line) and filtered data (dashed line) using a moving median and a complementary band-pass filters, with high-pass window size 861 and low-pass window size 21, for a 16 cm × 16 cm transducer plate.

Figure 8 shows the effects of the sensor drift and person rotations on the sensed signal for one of the sensors with a 16 cm × 16 cm plate attached (normalized to lay between 100 and 1 for the maximum and minimum frequency values, respectively). The rotation effects are significant, and will be discussed more in detail in the next section. The same figure also shows how a high-pass moving median filter attenuates the effects of the drift. The moving median filter is a low-pass filter whose output is calculated for each input data point *k* as the median of a subset (window) of input data points centered on input point *k* itself [22]:

(3) $$Y\left[k\right]=\mathrm{Median}\left(X\left(k-\frac{N-1}{2}:k+\frac{N-1}{2}\right)\right)$$

where *N* is the window size, *k* is the input point index, X[k] is the input signal, and Y[k] is the filtered output signal. The complexity of the median filter is O((N−1)/2)), which is suitable for on-sensor implementation. We use the filter as high-pass by subtracting its output from the input data, which attenuates mainly the DC drift.

The size of the filter window can be tuned based on the monitored activity. In this experiment, we used a window size of about 15 min (861 samples with 1 Hz sampling rate). This means that we lose track of a stationary person beyond 15 min, hence the localization algorithm should be able to compensate this.

#### 4.1.2. Short-Term Tracking Experiment

In this experiment we analyze the ability of our system to detect the path walked by a person inside the experiment room. Unlike the long-term experiment (see Section 4.1.1), the full data set was collected in the “short-term experiment” over a time interval of 2.5 min, in order to monitor a person who walks slowly but normally in the room. The objects did not change their position during the experiment, but realistic environmental noise from a fridge, a heater and several light bulbs was considered.

#### 4.1.3. Training Data Collection

We used the same floor grid as in the long-term experiment to control the movement of the person in the room along a pre-defined trajectory, as shown in Figure 6.

We kept a relatively low sampling rate (1 Hz) to lower the energy consumption of the sensors, while still being able to track the position of a person moving at about 1–2 km/h. This is enough to track the daily activities of an elderly person, which are typically made of slow movements and long stationary periods. We leave for future work the investigation of other sampling rates for different applications (e.g., surveillance and security). We believe that our sensors can also be used in those cases, most likely by changing the sampling rate and the filter characteristics.

In this experiment, the person stood in each position (Figure 6) for 10 s before moving to the next. We repeated this experiment 10 times, out of which 5 times the person was not wearing shoes in order to account for different body groundings (we did not notice any significant difference). Then we repeated the experiment another 10 times, all with the person having the body rotated by 90 degrees. These account for different body orientations, which can change the area and the distance body-sensor and significantly influence the transducer capacitance.

Thus, at the end of the training experiment we obtained 3200 four-tuple frequency samples from 20 data sets, each one with 10 samples/position for 16 body positions. The 20 data sets are made of: 10 data sets with the body rotated by 90 degrees and 10 data sets with the body facing the sensor, of which five are with shoes and five without shoes. We call this merged set of data the “Training Set” because we will use it to train the localization algorithms.

**Figure 9:**
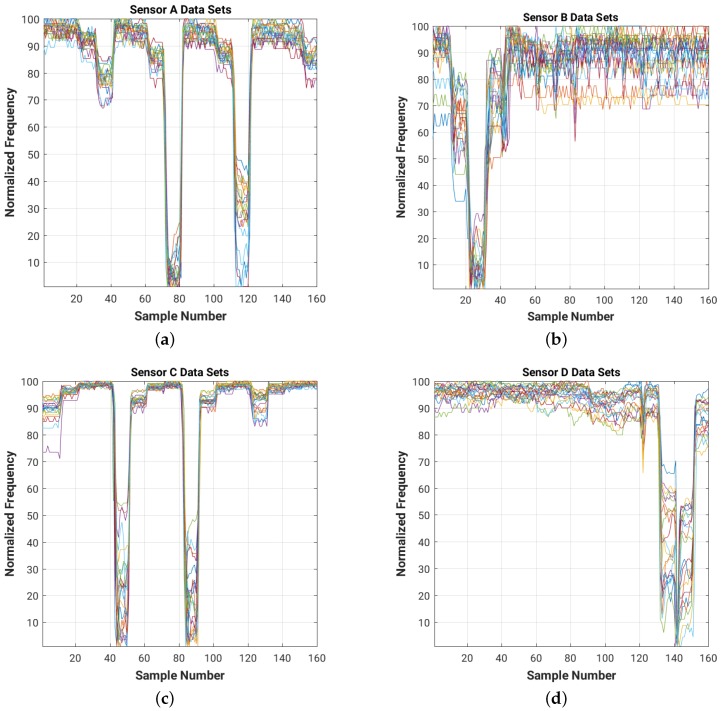
Normalized raw readings for each sensor ((**a**) Sensor A; (**b**) Sensor B; (**c**) Sensor C; (**d**) Sensor D), collected during the training experiment with 4 cm × 4 cm sensor plates. For each of the 16 positions in the room we collected 10 readings from each of the four sensors. Each color corresponds to one of the 20 training data sets.

For a quick visual comparison of the evolution of the frequency readings for each of the four sensors in the room for each of the 20 data sets, Figure 9 shows on the same graph the plots of the normalized frequency readings (with a value of 100 for the maximum frequency and of 1 for the minimum one).

**Figure 10:**
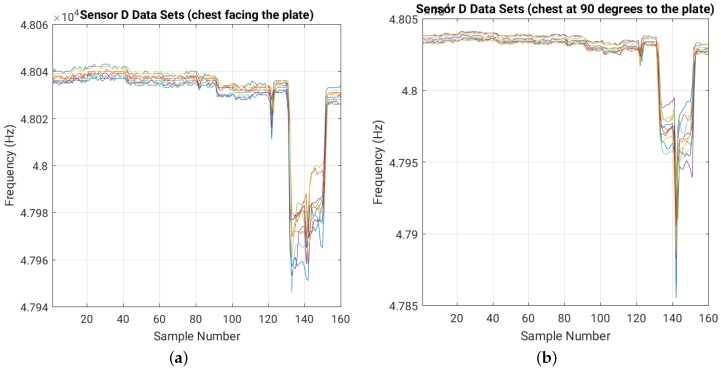
Sensor D raw training data sets using the 16 cm × 16 cm plate for (**a**) zero degree body-plate angle and (**b**) 90 degrees body-plate angle. For each of the 16 positions in the room we collected 10 readings. Each color corresponds to one of the 10 training data sets for each body orientation.

Figure 10 shows the effect of body orientation on sensor readings. We show just the results for sensor D with the 16 cm × 16 cm plate, since the other sensors and plate sizes show similar effects. We divided the 20 training data sets in two sets of 10 data sets each, based on the body orientation. We can see a similar sensor sensitivity for both body orientations. The large variations recorded when the person is closest to the sensor (e.g., around sample point 140 in both graphs in Figure 10) are due to the high sensitivity of the sensor to small body movements at short distance.

#### 4.1.4. Test Data Collection

**Figure 11:**
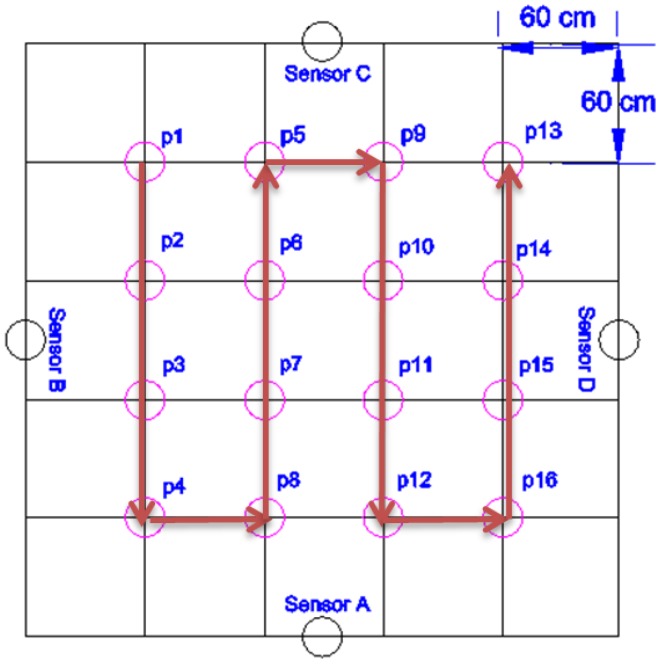
Movement trajectory of the person in room during Test Experiment (thick arrows).

In order to test our system, we performed a new experiment in which the person moved along a different trajectory (see Figure 11).

The set-up was the same as for the training experiment. We collected a total of five data sets, each with 10 samples for each of the 16 room positions for a total of 800 samples (5 data sets × 10 samples/position × 16 positions). We call this set the “Test Experiment” because we will use it to test the accuracy of the localization algorithms after they were trained using the data from the training experiment.

**Figure 12:**
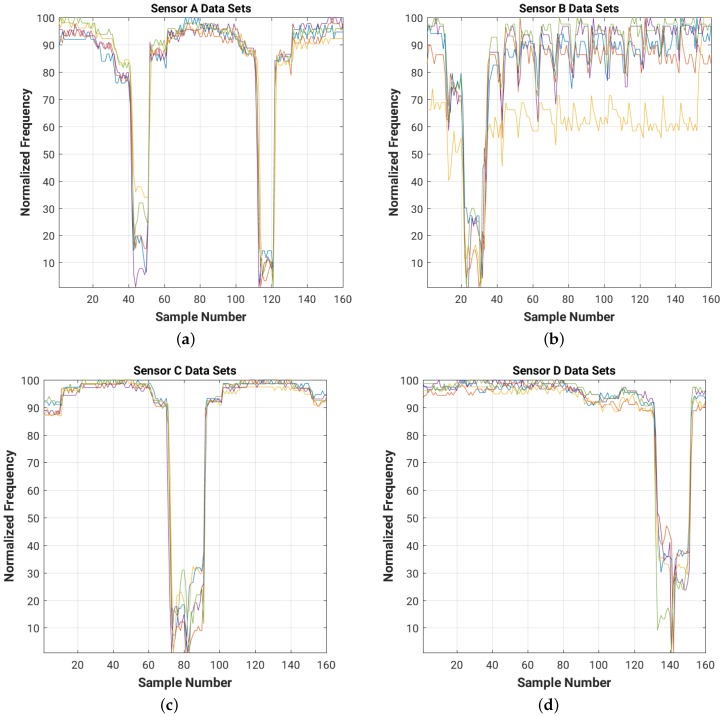
Normalized raw readings for each sensor ((**a**) Sensor A; (**b**) Sensor B; (**c**) Sensor C; (**d**) Sensor D), collected during the test experiment with 4 cm × 4 cm sensor plates. For each of the 16 positions in the room we collected 10 readings from each of the four sensors. Each color corresponds to one of the five test data sets.

For a quick visual comparison of the evolution of the frequency readings for each of the four sensors in the room for each of the 5 data sets, Figure 12 shows on the same graph the plots of the normalized frequency readings (with a value of 100 for the maximum frequency and of 1 for the minimum one).

It is worth mentioning that unlike in the training experiment, during the test experiment the person did not maintain a constant body orientation in all room positions. The reason is that we wanted to collect a more realistic movement scenario to test the system. Also, since during the training experiment we did not observe any significant difference while not wearing shoes, we performed the test experiment with the shoes on. The test experiment performed with 8 cm × 8 cm and 16 cm × 16 cm plates are reported in Appendix A.

Note that the data sets collected during both the training and test experiments have different DC components (shift in frequency level), which need to be filtered out. The high frequency noise (which can be easily observed in Figure 9 and Figure 11) needs to be attenuated as well in order to improve the overall signal-to-noise ratio (SNR) of the system.

### 4.2. Filter Block

As we discussed before, our signals are prone to different types of noise coming from, e.g., static discharge, home appliances or temperature and humidity variations. The noise effects are particularly significant at the far end of the sensor range, because there the capacitance variations induced by changes in body position are smaller. In fact, the sensor range is often limited by the noise level. As we will see in Section 5, the noise markedly degrades the accuracy of the localization algorithms unless it is properly attenuated using adequate filters.

**Figure 13:**
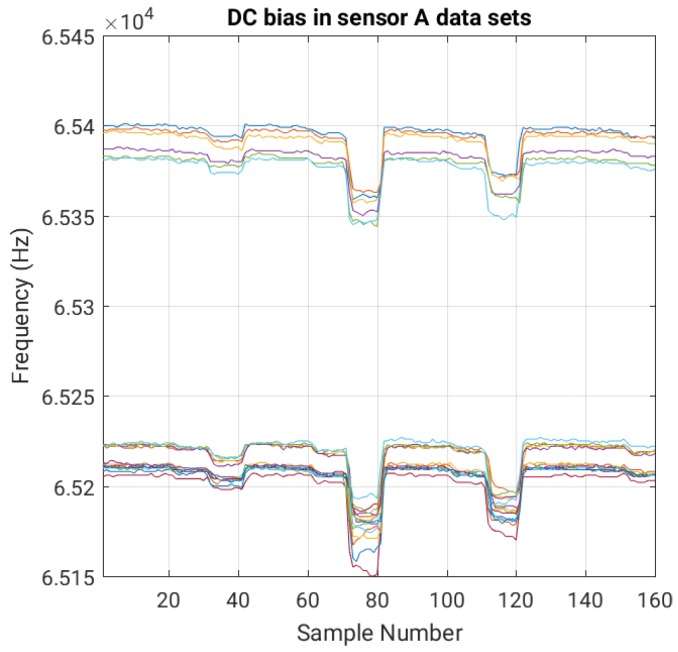
Different DC biases in the data sets of the training experiment for a sensor with a 4 cm × 4 cm plate. The measurements taken closer in time have similar biases (hence the clustering effects). Each color represents one of the 20 data sets in the training experiment.

The filtering block, implemented for now within the base station, attenuates the environmental noise to increase the effective sensitivity and accuracy of the system. The block consists of a digital low-pass and high-pass filter. The low-pass filter reduces the high frequency noise, e.g., coming from electric motors in appliances or light switches. The high-pass filter reduces the DC component of the signal. For instance, Figure 13 shows the DC biases in the training data sets. Each of the two clusters of data, the one close to the top and the one close to the bottom, contains data sets that were taken under very similar conditions and close to each other in time. On the other hand, the clusters themselves were separated by a large amount of time, during which static charge and other slowly varying conditions changed much more significantly.

### 4.3. Localization Algorithm Block

This block determines the position of the person within the monitored area using the filtered sensor data. We implemented this block using several well-known machine learning algorithms, as discussed in Section 5.2.

## 5. Optimization of the Filter and Localization Blocks

In this section we discuss how the blocks described in the previous section can be implemented to obtain the best localization results.

### 5.1. Filter Design

We used a moving median filter with a window of 5 as a low-pass filter to attenuate the high frequency noise and to remove a slowly varying DC bias from the signal. For high-pass filtering in a real deployment we would use the median filter discussed in Section 4.1.1. However, to keep the duration of the experiment within reasonable limits, we approximated its effect by subtracting the mean of the first 10 samples (where the person was far from all sensors) from the following 150 samples in each data set.

**Figure 14:**
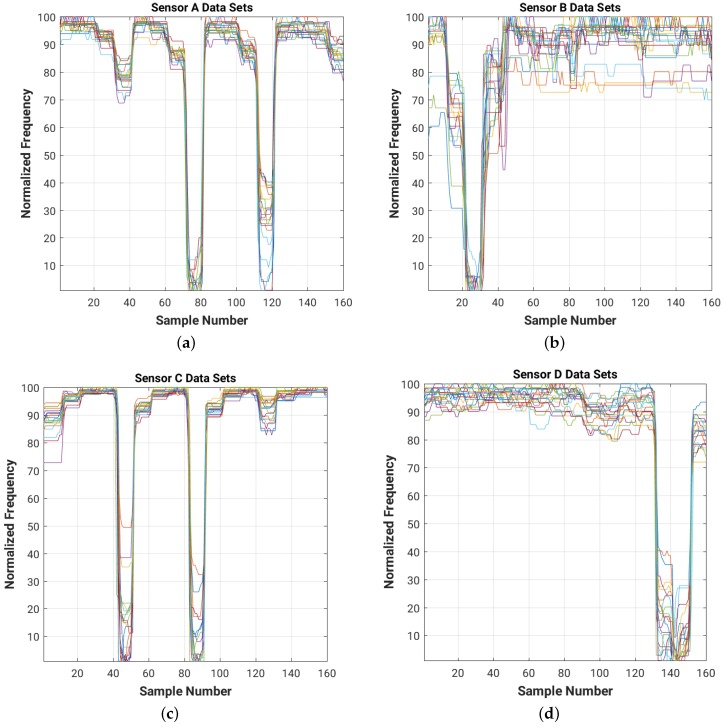
Normalized filtered readings for each sensor ((**a**) Sensor A; (**b**) Sensor B; (**c**) Sensor C; (**d**) Sensor D), collected during the training experiment with 4 cm × 4 cm sensor plates. Filter types: moving median low-pass with window size 5 and mean filter high-pass with windows size 10 (complementary band-pass filter).

**Figure 15:**
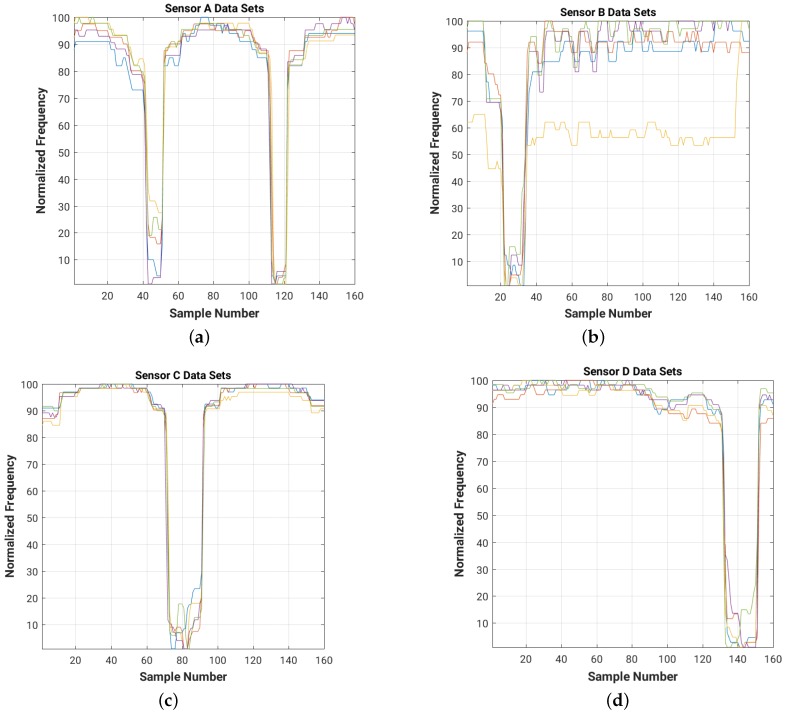
Normalized filtered readings for each sensor ((**a**) Sensor A; (**b**) Sensor B; (**c**) Sensor C; (**d**) Sensor D), collected during the test experiment with 4 cm × 4 cm sensor plates. Filter types: moving median low-pass with window size 5 and mean filter high-pass with window size 10 (complementary band-pass filter).

Figure 14 and Figure 15 show the results of filtering on the 4 cm × 4 cm sensor data (data for the 8 cm × 8 cm and 16 cm × 16 cm plate sizes are included in Appendix B). As we can see, the filters attenuate the high frequency noise (e.g., on sensor B data) and remove the DC bias of the signal.

After the filtering phase, we input the sensor data to the localization block, which determines the position of the person within the room.

### 5.2. Localization Algorithm Design

The localization algorithm operates in two modes: training mode and localization mode. The purpose of the training mode is to allow the localization algorithm to confidently associate a known position of the person in the room with specific values of the filtered sensor data, collected during the training experiment. The purpose of the localization mode is to infer the position of the person in the room from the filtered sensor data collected during the test experiment. As the person moves within the room, the localization algorithm tracks the position of the body.

Please note that in all experiments we provided the algorithms with filtered, but not normalized data. Data normalization was used only to simplify the visual comparison in some figures.

We use three types of standard supervised classification algorithms, namely: k-Nearest-Neighbors (k-NN), Naïve Bayes (NB), and Support Vector Machines (SVM).

#### 5.2.1. k-Nearest-Neighbors

**Figure 16:**
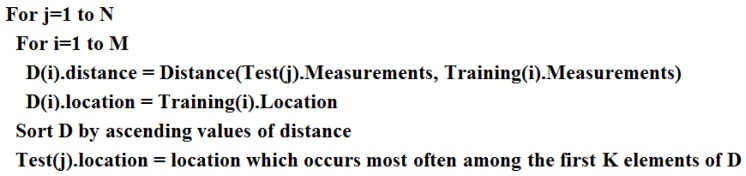
k-Nearest-Neighbors (k-NN) classifier operation in pseudo-code.

k-NN classification is a non-parametric approach which does not need to know in advance how the data is distributed in order to perform its task. The k-NN classifier is typically based on the Euclidean distance between a test sample and the training samples [23]. The operation of the algorithm is shown in pseudo-code in Figure 16.

The algorithm takes two inputs:

- “Training” is a vector of M records, where each element has:
  – A tuple of measurements (in our cases, a 4-tuple of frequencies, since we have four sensors)
  – A location label
- “Test” is a vector of N records, where each element has:
  – A tuple of measurements, like in the training set
  – A location label (initially undefined)

The output consists of the assignment of a location label to each test vector element.

For each test data point, the algorithm computes the Euclidean distance (which in general could use different weights for different sensors; in this case we use uniform weights) between the test point and all the data points in the training set. According to these distances, the algorithm then selects the location in the training set that occurs most frequently among the closest *k* to the test data point. The recommended number of neighbors *k* is usually equal to the square root of the number of observations [24]. For our training experiment this means $k=\sqrt{3200}\approx 56$.

Note that during the test phase the classifier needs to have all training samples in memory to calculate the distances between them and a test sample. So both its complexity to classify a new test sample and its memory occupation are O(M·K), where *M* is the number of samples in the training set and *K* is the number of features. In our case K=4, because we have four streams of data coming from the four room sensors. This means that the k-NN localization algorithm is fairly expensive at classification time.

#### 5.2.2. Naïve Bayes

Naïve Bayes (NB) is a parametric supervised classification algorithm. In our case, during the training phase it calculates the statistical mean and standard deviation of the data points for each room position. During the test phase, it first estimates the probability for a given data point in the test set to belong to each room position, then it assigns this data point to the room position with the highest probability.

Note that finding a suitable kernel distribution during the training phase can be very time consuming, because it requires to find a distribution kernel which fits best all our training sensor data. However, this speeds up dramatically the analysis during the test phase. In other words, NB is slower than k-NN during training, but it has much lower memory and time requirements than k-NN during the test phase (O(K) complexity and memory size [25]). This is perfectly acceptable, because the training phase is performed only once and does not need to be done in real time.

#### 5.2.3. Support Vector Machine

Support Vector Machine (SVM) is a non-parametric classifier designed for binary classification (i.e., between only two classes), but it can be extended to more classes with a simple generalization.

SVM basic idea is to find a geometrical separator between the available classes (the 16 locations in our case) in a *k*-dimensional space, where *k* is the number of features or attributes (the four sensors in our case). This separator is defined as a kernel function that can be linear, polynomial or, most commonly, Gaussian in shape. For the training phase, we used a multi-class generalization of SVM with a Gaussian kernel [26]. For the test phase, we labeled the test samples based on the fitted SVM kernel function. SVM is significantly slower than NB and k-NN during training because it has $O(M^{2}\;\cdot \;K\;\cdot \;C)$ complexity [27] (where *C* is the number of classes, 16 in our case). Its complexity during the test phase is low, similar to that of NB.

## 6. Results and Discussion

In this section we report the average and standard deviation of the localization error, the recall, the precision, and the path detected by the localization algorithms described above, for sensors with 4 cm × 4 cm, 8 cm × 8 cm, and 16 cm × 16 cm plates.

We include the results of the 1-NN algorithm only as reference to show how choosing the proper number of neighbors (56 in our case) can significantly affect the localization results.

In general, the k-NN algorithm does not make any assumption on noise type and treats all samples as equal. NB assumes Gaussian noise and gives less importance to outliers. The SVM algorithm may end up using too many classes, with boundaries that may not be well captured by its Gaussian separator.

### 6.1. Distance Error

The estimated error is calculated as the distance between the actual position of the person in the room and the position estimated by the system. The error is calculated for each test sample, then it is averaged over all *N* samples in the test set (the use of other norms does not change the results significantly). We also report the standard deviation (STD) of the error.

**Figure 17:**
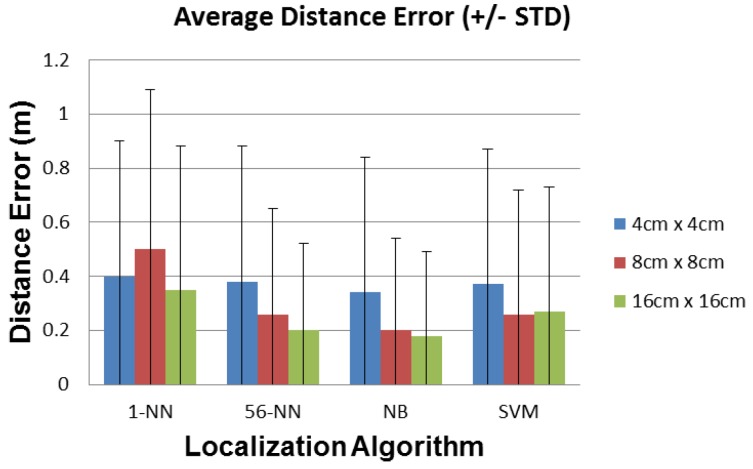
Average distance error (±STD) for each localization algorithm and sensor plate size.

Figure 17 shows the average estimated position error for various localization algorithms and plate sizes. NB has generally the best overall performance in terms of mean distance error (also considering the STD) for all plate sizes. It has less than 20 cm average distance error for the 8 cm × 8 cm and 16 cm × 16 cm plates. 1-NN has the poorest performance, with 50 cm average distance error using the 8 cm × 8 cm plate and also the most noticeable non-monotonic behavior with respect to plate area.

### 6.2. Recall and Precision

Recall and precision are calculated using Equations (4) and (5), respectively

(4) $$\mathrm{Recall}\;(\%)=\frac{\mathrm{True}\;\mathrm{Positive}}{\mathrm{True}\;\mathrm{Positive}+\mathrm{False}\;\mathrm{Negative}}\times 100$$

(5) $$\mathrm{Precision}\;(\%)=\frac{\mathrm{True}\;\mathrm{Positive}}{\mathrm{True}\;\mathrm{Positive}+\mathrm{False}\;\mathrm{Positive}}\times 100$$

where True Positive is the number of samples collected and correctly classified as a specific position, False Negative is the number of samples collected in a specific position but classified incorrectly, and False Positive is the number of samples collected in other positions but classified incorrectly as a specific position.

**Figure 18:**
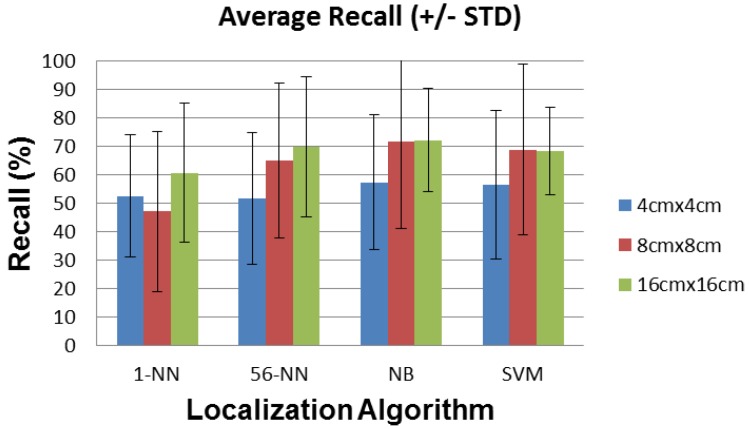
Average recall (±STD) for each localization algorithm and sensor plate size.

**Figure 19:**
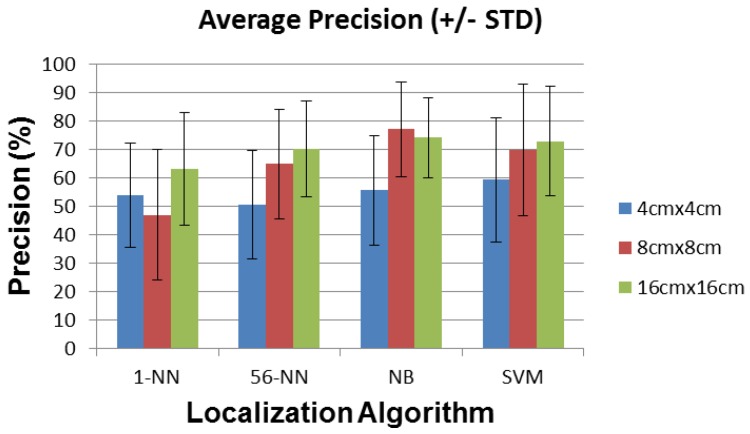
Average precision (±STD) for each localization algorithm and sensor plate size.

Figure 18 and Figure 19 show the recall and precision of the localization algorithms for all plate sizes. NB performs again best among all localization algorithms, reaching more than 70% recall and precision on average with the 8 cm × 8 cm and the 16 cm × 16 cm plates. Sensor performance with the 4 cm × 4 cm plates is limited mostly by the reduced range compared to room dimensions. The sensors with 8 cm × 8 cm and 16 cm × 16 cm plates have adequate ranges and are limited mostly by their noise floor. Since the sensor plates also behave as patch antennas, they may inadvertently tune to (and thus amplify) different sources of environmental noise when changing their dimensions. This may degrade their SNR and explain the similar results obtained for sensors with 8 cm × 8 cm and 16 cm × 16 cm plate sizes.

The performance of the 1-NN algorithm changes again non-monotonically with plate size, unlike 56-NN, which shows a constant improvement for both recall and precision. NB also shows a slight non-monotonicity for the 16 cm × 16 cm vs. the 8 cm × 8 cm plate size. However, this difference is well within the ±STD interval, so it is most likely not significant.

**Figure 20:**
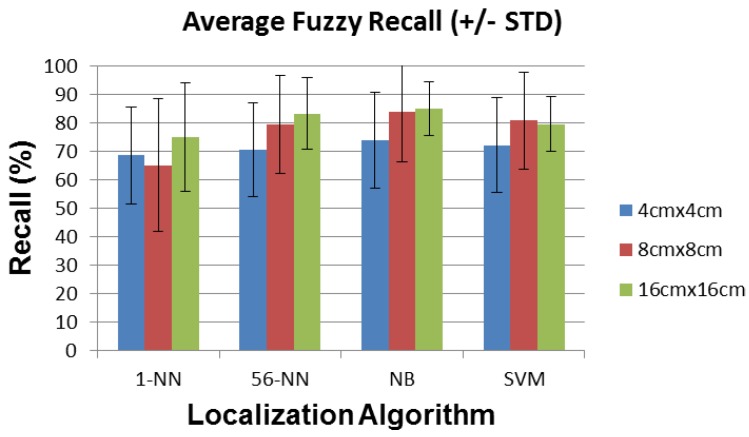
Average fuzzy recall (±STD) for each localization algorithm and sensor plate size.

**Figure 21:**
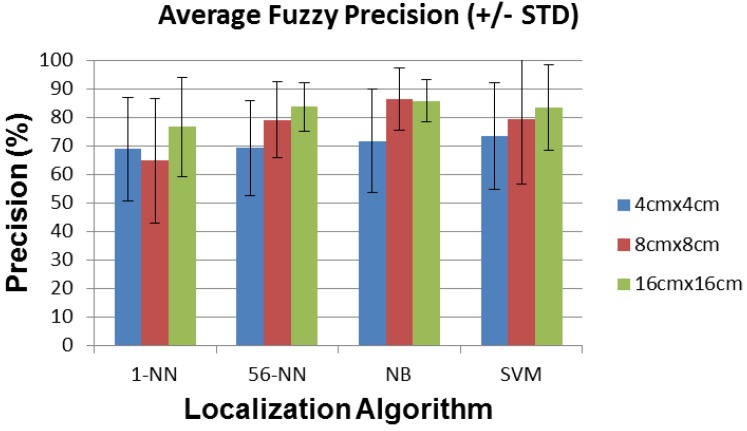
Average fuzzy precision (±STD) for each localization algorithm and sensor plate size.

Note that precision and recall defined as in Equations (4) and (5) do not take into account the fact that a small distance error is better than a large distance error for our application. In order to account for this, we introduce a weighting mechanism with a weight of 1 for the exact detected position, 1/2 for the orthogonally adjacent detected position and 1/3 for the diagonally adjacent detected position. The resulting fuzzified recall and precision parameters shown in Figure 20 and Figure 21 are significantly improved.

### 6.3. Path (Trajectory) Detection

**Figure 22:**
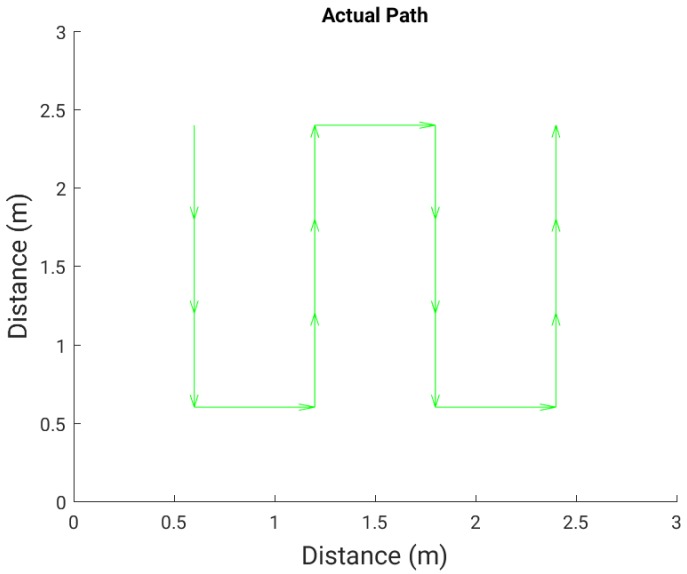
The actual path walked by the person in the room.

**Figure 23:**
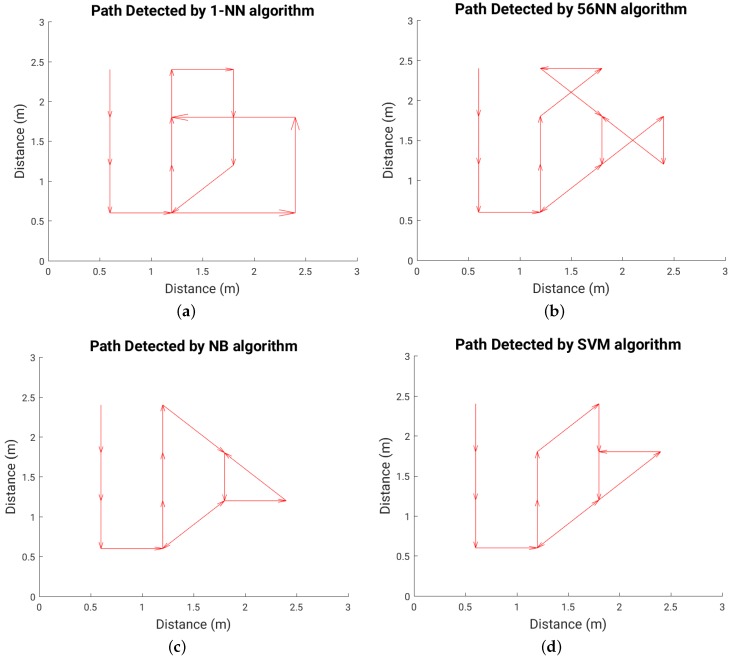
Detected path for each localization algorithm in the case of 4 cm × 4 cm plate; (**a**) 1-NN algorithm; (**b**) 56-NN algorithm; (**c**) NB algorithm; (**d**) SVM algorithm.

**Figure 24:**
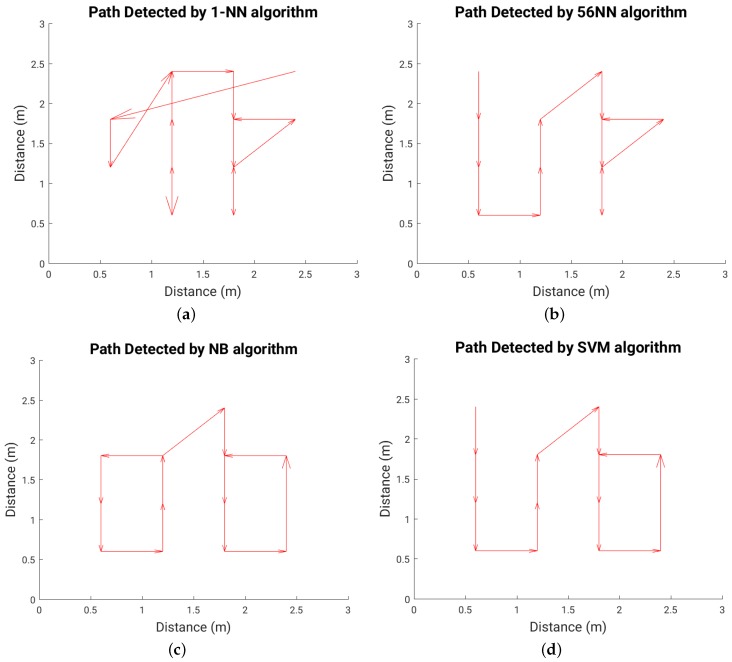
Detected path for each localization algorithm in the case of 8 cm × 8 cm plate; (**a**) 1-NN algorithm; (**b**) 56-NN algorithm; (**c**) Naïve Bayes (NB) algorithm; (**d**) Support Vector Machines (SVM) algorithm.

**Figure 25:**
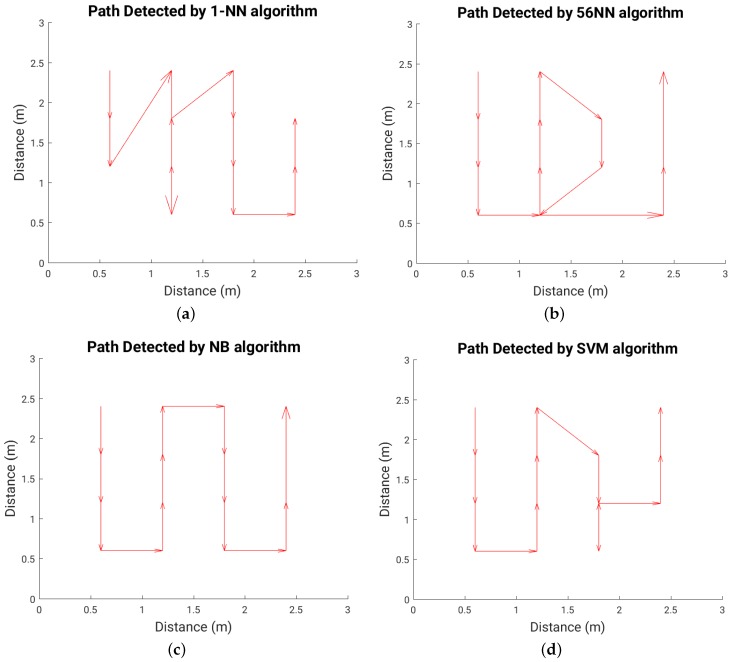
Detected path for each localization algorithm in the case of 16 cm × 16 cm plate; (**a**) 1-NN algorithm; (**b**) 56-NN algorithm; (**c**) NB algorithm; (**d**) SVM algorithm.

Figure 22 shows the path walked during the test experiment, while Figure 23, Figure 24 and Figure 25 show the paths detected by the various localization algorithms for different plate sizes (the arrow size is proportional to the length of each movement vector). We can see a monotonic improvement across all localization algorithms with the plate size. Also, a k-NN algorithm with more neighbors detects better the path for all plate sizes. The SVM algorithm shows a good path detection with just a small deviation in the case of both 8 cm × 8 cm and 16 cm × 16 cm sensor plates. NB detects best the path overall, and for the 16 cm × 16 cm plate it detects correctly the full walked path (Figure 25c).

## 7. Conclusions and Future Work

Unobtrusive passive tag-less privacy-aware indoor human location estimation adds a great value to assisted living scenarios. In this article we introduce a tag-less passive localization system based on capacitive sensing that can track the position of a person in a 3 m × 3 m room with about 20 cm error and better than 70% recall and precision (better than 80% fuzzified recall and precision). We compare the experimental results in terms of precision, recall, average distance error, and detected walking path for various sensor sizes (4 cm × 4 cm, 8 cm × 8 cm and 16 cm × 16 cm) and several localization algorithms. Generally all figures of merit improve significantly with plate area. The NB algorithm achieves perfect path detection using the 16 cm × 16 cm plates.

The results, based on a set of realistic experiments, are very encouraging, because they achieve excellent accuracy with simple algorithm training. We discuss the various kinds of noise that affect the sensor signal during a long-term experiment and also how signal filtering can reduce the localization error for long-term position tracking.

We plan to deploy our system for long-term position tracking and also to explore data fusion techniques. For example, PIR sensors could be used as an error correction mechanism for the system because they are sensitive to sources of noise (e.g., heat) to which the capacitive sensors are immune, and vice-versa.

We will also experiment with different sampling frequencies and sensor positioning strategies, as well as analyze the privacy and security aspects of the approach. We also want to analyze how to use the technique to locate, and possibly discriminate between, more than one person in a given room.

## Figures and Tables

**Figure A1:**
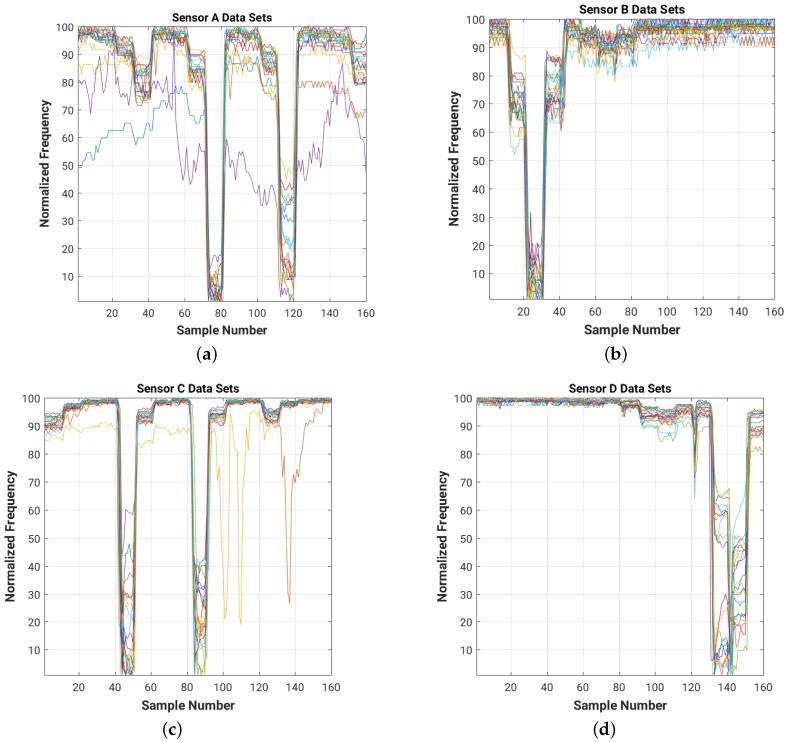
Normalized raw readings for each sensor ((**a**) Sensor A; (**b**) Sensor B; (**c**) Sensor C; (**d**) Sensor D), collected during the training experiment with 8 cm × 8 cm sensor plates. For each of the 16 positions in the room were collected 10 readings from each of the four sensors. Each color corresponds to one of the 20 training data sets.

**Figure A2:**
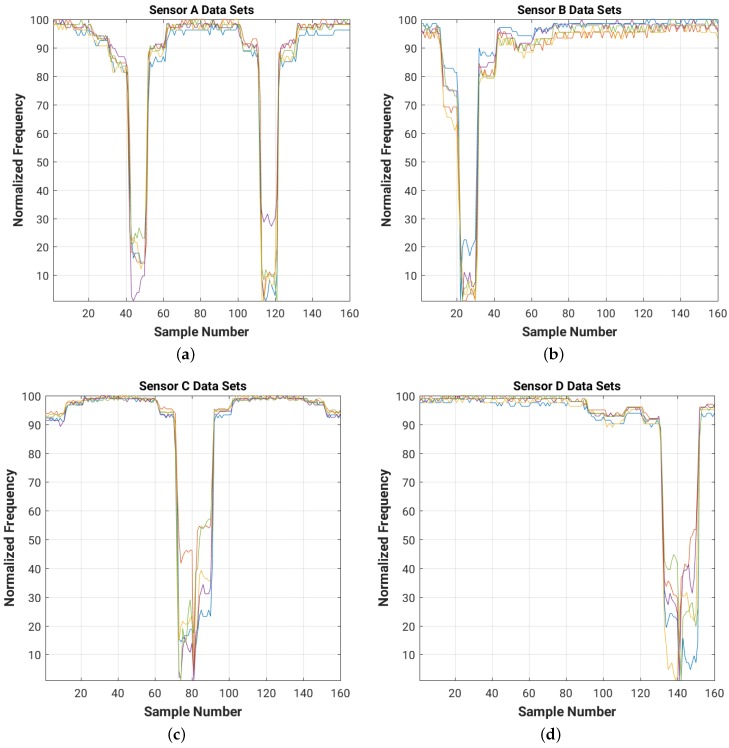
Normalized raw readings for each sensor ((**a**) Sensor A; (**b**) Sensor B; (**c**) Sensor C; (**d**) Sensor D), collected during the test experiment with 8 cm × 8 cm sensor plates. For each of the 16 positions in the room were collected 10 readings from each of the 4 sensors. Each color corresponds to one of the five test data sets.

**Figure A3:**
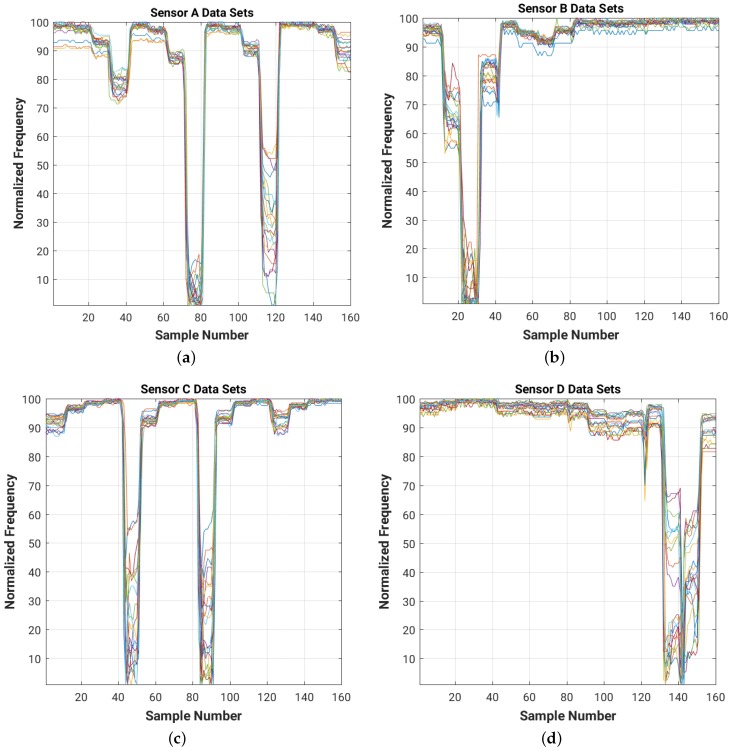
Normalized raw readings for each sensor ((**a**) Sensor A; (**b**) Sensor B; (**c**) Sensor C; (**d**) Sensor D), collected during the training experiment with 16 cm × 16 cm sensor plates. For each of the 16 positions in the room were collected 10 readings from each of the 4 sensors. Each color corresponds to one of the 20 training data sets.

**Figure A4:**
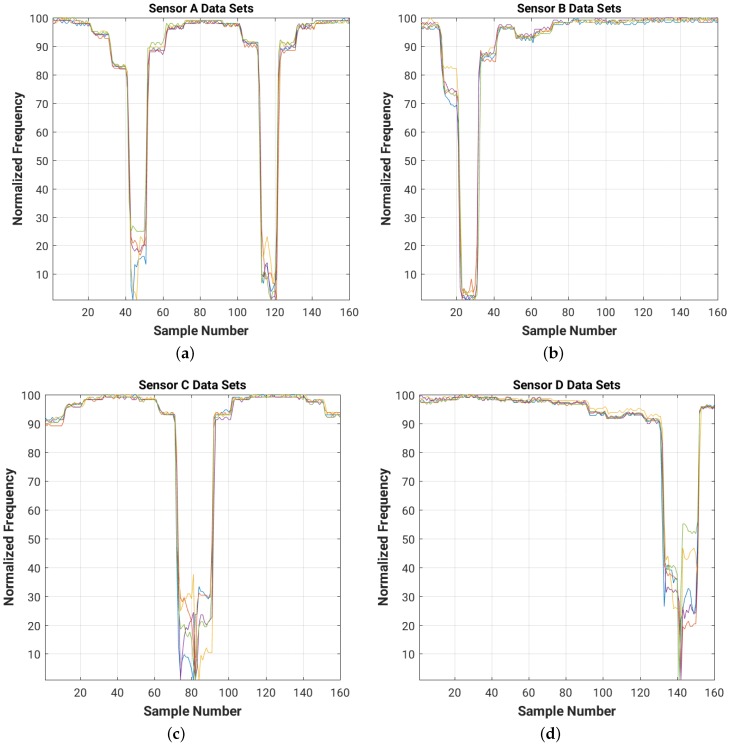
Normalized raw readings for each sensor ((**a**) Sensor A; (**b**) Sensor B; (**c**) Sensor C; (**d**) Sensor D), collected during the test experiment with 16 cm × 16 cm sensor plates. For each of the 16 positions in the room were collected 10 readings from each of the four sensors. Each color corresponds to one of the five test data sets.

**Figure B1:**
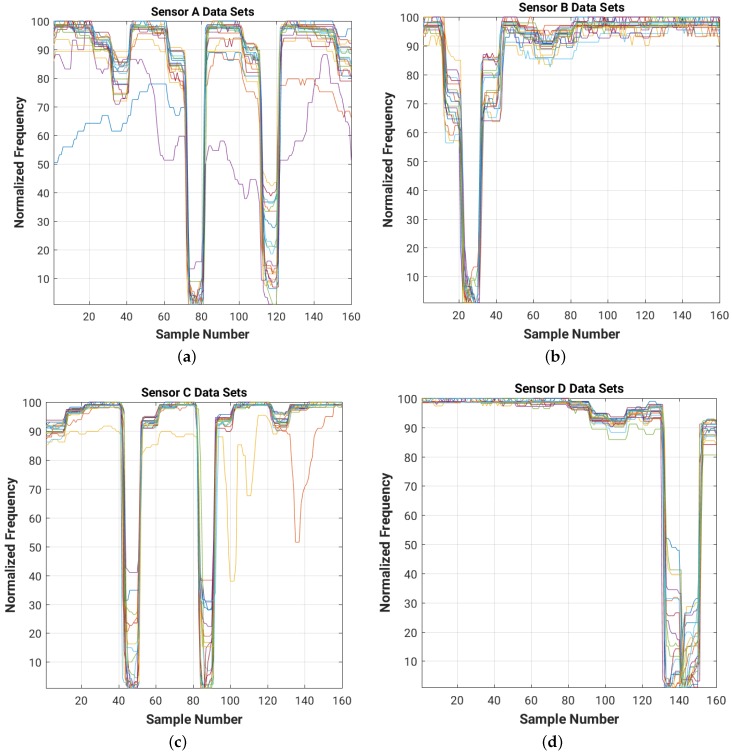
Normalized filtered readings for each sensor ((**a**) Sensor A; (**b**) Sensor B; (**c**) Sensor C; (**d**) Sensor D), collected during the training experiment with 8 cm × 8 cm sensor plates. Filter types: moving median low-pass with window size 5 and mean filter high-pass with windows size 10 (complementary band-pass filter).

**Figure B2:**
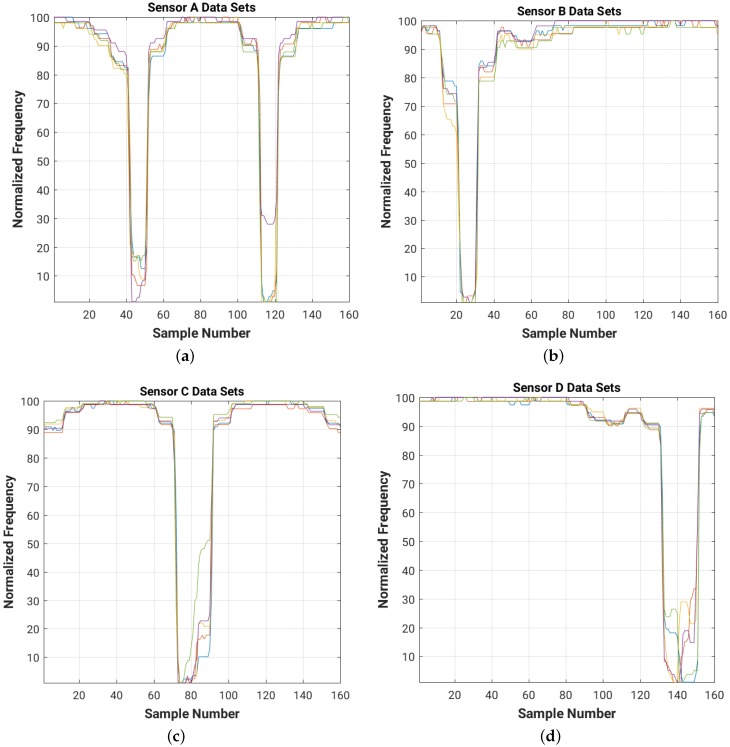
Normalized filtered readings for each sensor ((**a**) Sensor A; (**b**) Sensor B; (**c**) Sensor C; (**d**) Sensor D), collected during the test experiment with 8 cm × 8 cm sensor plates. Filter types: moving median low-pass with window size 5 and mean filter high-pass with window size 10 (complementary band-pass filter).

**Figure B3:**
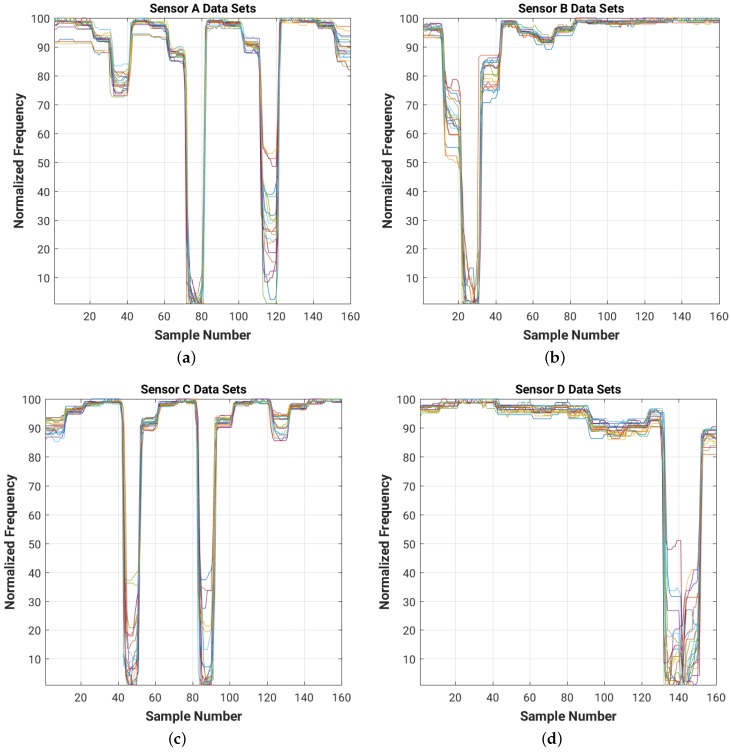
Normalized filtered readings for each sensor ((**a**) Sensor A; (**b**) Sensor B; (**c**) Sensor C; (**d**) Sensor D), collected during the training experiment with 16 cm × 16 cm sensor plates. Filter types: moving median low-pass with window size 5 and mean filter high-pass with windows size 10 (complementary band-pass filter).

**Figure B4:**
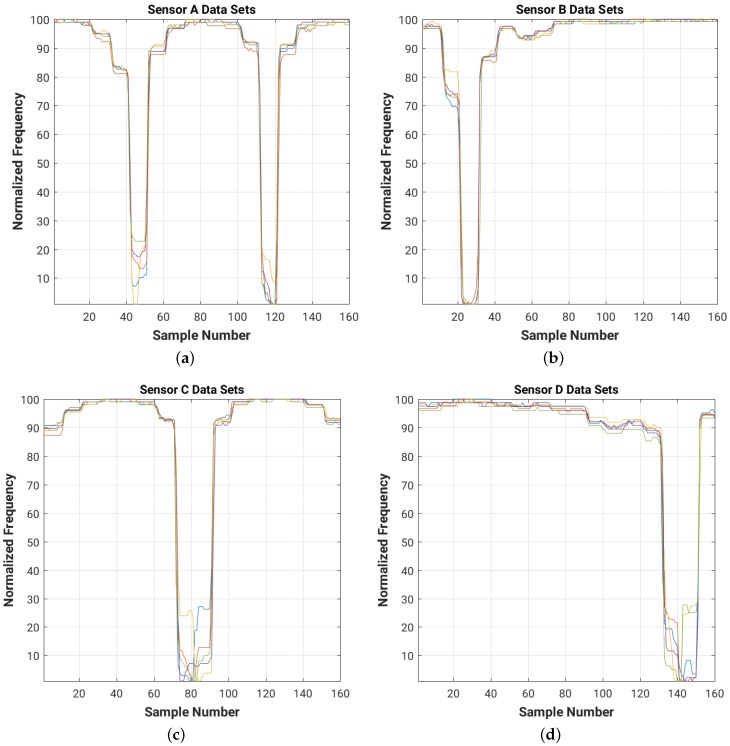
Normalized filtered readings for each sensor ((**a**) Sensor A; (**b**) Sensor B; (**c**) Sensor C; (**d**) Sensor D), collected during the test experiment with 16 cm × 16 cm sensor plates. Filter types: moving median low-pass with window size 5 and mean filter high-pass with window size 10 (complementary band-pass filter).

## Supplementary Materials

The following are available online at http://www.mdpi.com/1424-8220/16/9/1448/s1.
